# Analysis of Three-Dimensional Morphological Differences in the Mandible between Skeletal Class I and Class II with CBCT Fixed-Point Measurement Method

**DOI:** 10.1155/2021/9996857

**Published:** 2021-05-07

**Authors:** Qiang Dong, HaoYu Shi, Qi Jia, Yueyi Tian, Keqian Zhi, Lu Zhang

**Affiliations:** ^1^Department of Stomatology, The Affiliated Hospital of Qingdao University, Qingdao, 266003 Shandong, China; ^2^School of Stomatology of Qingdao University, Qingdao, 266003 Shandong, China; ^3^The Conversationalist Club, School of Stomatology, Shandong First Medical University, Tai'an, 271016 Shandong, China

## Abstract

This study was aimed at determining the three-dimensional differences in the mandible morphology between skeletal class I and II patients, at exploring the pathogenic mechanisms and morphological characteristics of skeletal class II, and at providing clinical references. The subjects were assigned to two groups according to the size of ANB angle: skeletal class I (2° < ANB angle < 5°) and skeletal class II (5° < ANB angle < 8°). After cone-beam computed tomography (CBCT) scanning, 31 landmarks and 25 measurement items were determined by In Vivo Dental 5.1 software (Anatomage, CA) for statistical analysis. The results were as follows: Co-Go, Go-Me, and CdM-CdD in skeletal class II cases were smaller than those in skeletal class I, and GoR-Me-GoL, GoR-Me-CoL, and, Ig-Men were larger than those in skeletal class I cases. In conclusion, there were significant differences in the three-dimensional morphology of the mandible between skeletal class I and class II patients. The vertical growth of the ramus, the horizontal growth of the mandibular body, and the condyle in skeletal class II patients were smaller than those in skeletal class I cases. In skeletal class II, the growth of the anterior part of the mandible in the vertical direction was larger than that in skeletal class I, and the shape of the mandible was more extended.

## 1. Introduction

Skeletal class II malocclusion is a common jaw deformity in the clinic, with a prevalence rate of approximately 20%. It is characterized by abnormal mandibular morphology and abnormal sagittal position between the maxilla and mandible [1]. The mandible is an important component of the craniofacial complex, in the lower one-third of the face. Genetic and environmental factors impact mandibular size and morphology easily, which play an important role in the esthetic appearance and function of the maxillofacial region. Uncoordinated facial contour caused by the mandible is one of the most common causes for patients seeking orthodontic treatment [2]. Therefore, orthodontists should pay close attention to the mandibular size, morphology, position, and rotation angle to select an appropriate treatment plan [3].

CBCT has the advantages of fast scanning, low effective radiation dose, simple procedural steps, etc. Its accuracy of the three-dimensional reconstruction is high, with a true one-to-one measure of craniofacial anatomy. It can be directly measured on three-dimensional reconstructed images, similar to the anatomical measurement [4–7]. The extensive application of CBCT in the clinic and the increasingly optimized third-party image processing software significantly improve the measurement of craniomaxillofacial structures, providing more accurate and comprehensive information [8].

The mandible is a three-dimensional structure with an irregular shape. Two-dimensional images cannot fully and accurately reflect the actual situation of three-dimensional structures. Thus, in clinical orthodontics, three-dimensional reconstruction and measurement analysis of the mandible is of great significance [9]. Despite some achievements in the three-dimensional morphology of the mandible, the data are still relatively scarce. This study was aimed at determining the three-dimensional mandibular morphology in patients with skeletal class I and class II malocclusion, at exploring the differences between them, and at providing a reference for the pathogenesis, classification, and treatment of skeletal class II malocclusion.

## 2. Materials and Methods

### 2.1. Case Selection

The total sample (72 volunteers) was obtained at the Orthodontic Department, Affiliated Hospital of Qingdao University.

The inclusion criteria were as follows: (1) Han nationality aged 18-25 years, (2) facial symmetry, (3) complete dentition, and (4) clear and complete CBCT images.

The exclusion criteria were as follows: (1) history of oral and maxillofacial trauma, (2) history of orthodontic or orthognathic treatment, (3) severe soft and hard tissue lesions in the maxillofacial region, (4) systemic diseases, and (5) blurred, distorted, and overlapping CBCT images with affects, affecting the identification and measurement.

The subjects were divided into class I or class II skeletal patterns, according to the ANB angle.

*Skeletal class I (*2° < *ANB* *angle* < 5°): 36 cases, including 18 males and 18 females.

*Skeletal class II (*5° < *ANB* *angle* < 8°): 36 cases, including 18 males and 18 females.

### 2.2. Scanning and Reconstruction

The CBCT images of the patients were captured by a CBCT unit (Pax-Zenith3d, EWOO-VATECH, Korea). The subjects' facial midline was adjusted perpendicular to the floor, the head position was fixed, and the lips were occluded and closed naturally, breathing calmly without swallowing. The source data of volunteers were obtained and stored in a DICOM format: scanning range: wide field; scanning conditions: tube voltage 90 kV and tube current 4 mA. DICOM files were imported into the same orthodontic workstation computer installed with In Vivo Dental 5.1 software to reconstruct the image data.

### 2.3. Determination of Mark Points and Measurement Items

Ideal landmarks should be anatomic landmarks that are easy to locate and relatively stable, reflecting the morphological characteristics of the mandible. Through literature review and analysis, 31 commonly used three-dimensional measurement landmarks of the mandible were selected. It includes 5 coordinate system points, 6 single points, and 20 paired points (Table 1).

According to the relevant literatures, a total of 25 line distance and angle measurements were selected to describe the morphological characteristics of the mandible, of which 10 measurements were used to describe the characteristics of the mandibular ramus, and 8 measurements were used to describe the characteristics of condyle and coracoid process [10–12]. The line distance is measured in “mm” and the angle is measured in “°” (Table 2).

### 2.4. Three-Dimensional Items

**Figure 1:**
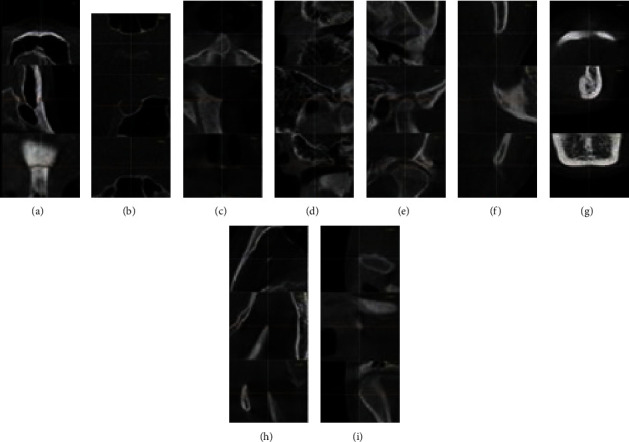
The 3D positioning maps of (a) N, (b) S, (c) ANS, (d) Po-R, (e) Co-L, (f) Go-L, (g) Men, (h) Cc-R, and (i) CdDR.

A 3D analysis module of In Vivo Dental 5.1 software was used to fix 3D points. Turn on the “slice locator” function to display the position of the marked points in the cross section, sagittal plane, and coronal plane. Adjusting the position by moving three-dimensional landmarks on the three sections and combining with the three-dimensional model of the head, the accuracy and reliability of the three-dimensional fixed point can be improved. The 3D positioning maps of some landmark points are shown in Figure 1.

### 2.5. Three-Dimensional Measurement

**Figure 2:**
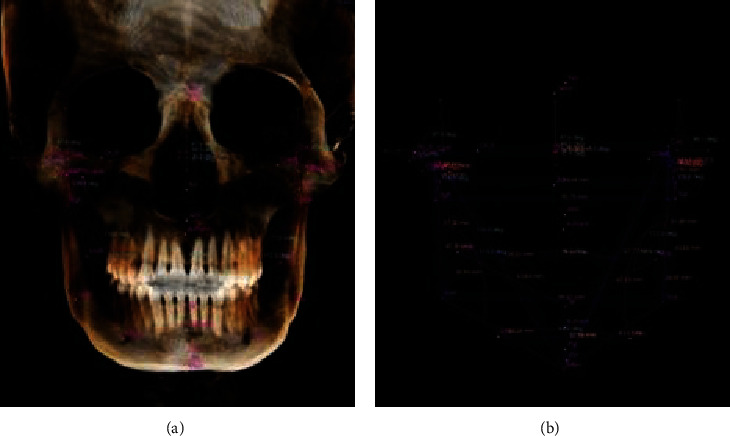
Three-dimensional measurement result of (a) angle and (b) line distance (anterior view).

**Figure 3:**
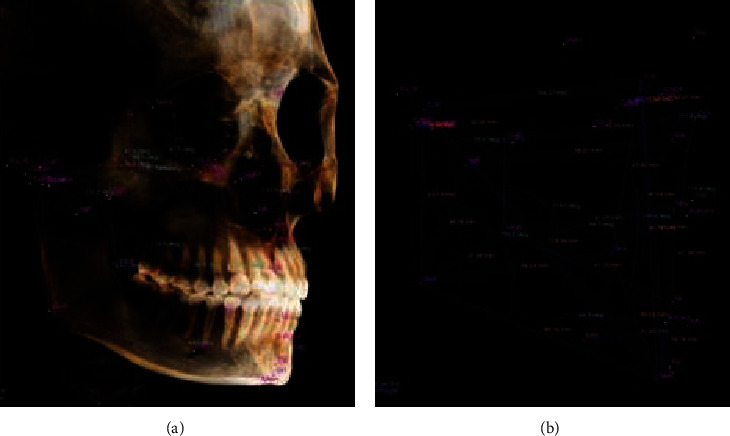
Three-dimensional measurement result of (a) angle and (b) line distance (45° lateral view).

**Figure 4:**
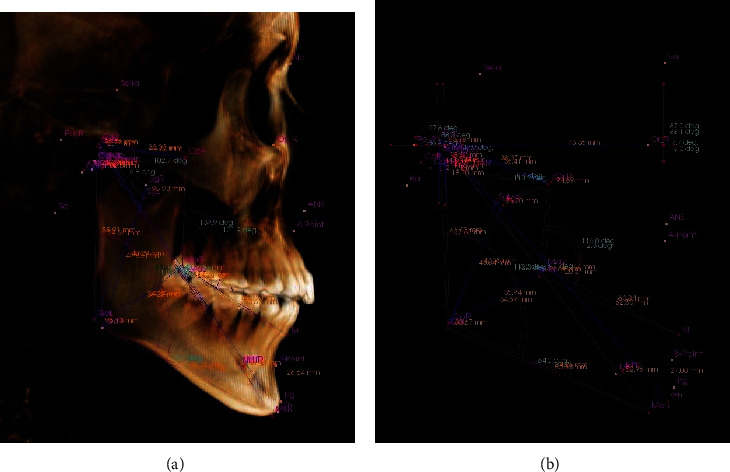
Three-dimensional measurement result of (a) angle and (b) line distance (lateral view).

25 measurement items were set in the 3D analysis module of In Vivo Dental 5.1 software. After the 3D fixed point was completed, set line distance and angle measurement values were automatically obtained. Each patient was measured twice by the same person with an interval of one week, and the average value of the two measurements was taken as the 3D measurement result (Figures 2–4).

### 2.6. Statistical Analysis

SPSS (IBM, USA) was used for statistical analysis. The statistical differences between class I and class II, between class I men and class II men, and between class I women and class II women were compared. Shapiro-Wilk test was used to determine whether the data were in accordance with normal distribution, and independent sample *t*-test was used for the difference. *P* < 0.05 indicates a significant difference.

## 3. Result

### 3.1. Skeletal Class I and Class II

There were six items with statistical difference between skeletal class I and skeletal class II (Table 3). Co-Go, Go-Me, and CdM-CdD of skeletal class II were smaller than those of skeletal class I. Ig-Men, GoR-Me-GoL, and CoR-Me-CoL were larger than those of skeletal class I. There was no significant difference in other measurement items.

### 3.2. Skeletal Class I and Class II in Male

Go-Me and CdM-CdD of skeletal class II were smaller than those of skeletal class I. Ig-Men and GoR-Me-GoL were larger than those of skeletal class I (Table 4).

### 3.3. Skeletal Class I and Class II in Female

Go-Me, Co-Go, CdM-CdD, and CdA-CdP in skeletal class II were smaller than those in skeletal class I (Table 5).

## 4. Discussion

### 4.1. Advantages of Three-Dimensional Measurement in the Mandible

Three-dimensional measurement technology includes laser scanning technology, structured light scanning technology, X-ray technology, and spiral CT scanning technology, which is widely used in clinical and scientific researches [9, 13–16]. The early study of the mandible was mainly based on lateral cephalogram. Technological advances in CBCT appear to offer significant advantages in both quality and quantity of data representing true anatomy [17, 18]. We can analyze morphological structure and position of the mandible in three-dimensional direction by using CBCT, so as to better refer to the scientific research and clinical work in the field of orthodontics.

Accurate fixed location of anatomical landmarks is the premise of obtaining reliable analysis results. The three-dimensional landmarks selected in this experiment are supported by previous literatures [19]. They are easy to determine, with rare variation, which can truly reflect the shape and structure of the mandible [10–12]. Point Co and other paired points are independent and related to each other. In the traditional two-dimensional measurement method, the images of these points are the superposition or the mean value of the two, which ignore part of the superposition or the mandible, causing loss of mandibular information. In this experiment, the paired points were marked separately to increase the transverse line distance of the mandible, such as GoR-GoL, MfR-MfL, and CdM-cDd, which reflected the horizontal characteristics of the mandible and obtained more comprehensive information to describe the characteristics of the mandible.

The three-dimensional measurement makes it possible to measure the items that cannot be measured on a two-dimensional plain film. It can not only measure the line distance and angle on the same plane but also measure the distance between the point and plane, the angle of project between line and plane, etc. which greatly improves the richness of measurement. For example, in this experiment, the angle between the ramus and horizontal plane projected on the median sagittal plane represents its anteroposterior inclination (-Sag), and the angle between the ramus and horizontal plane projected on the coronal plane represents its mesial and distal inclination (-Cor).

In the future, a three-dimensional cephalometric method can be established to help orthodontists get better diagnosis and treatment plan. However, it should be noted that the significance of many measurement items has changed after the transformation from two-dimensional measurement to three-dimensional measurement. The traditional cephalometric measurement is the result of the projection of the structure to the median sagittal plane, which reflects a single-dimensional relationship. In the three-dimensional measurement, most of the measurement items are not in one plane, which often reflect the relationship between two or even three dimensions [20]. The reference significance and normal value of each measurement item in three-dimensional cephalometry need to be further studied [20].

### 4.2. The Clinical Significance of Mandibular Morphology Difference between Skeletal Class I and Skeletal Class II

Skeletal class II malocclusion is a common malocclusion in the clinic, characterized by deep overbite of the anterior teeth, open lips, exposed teeth, maxillary protrusion, and mandibular retrusion, seriously affecting the facial appearance and chewing functions [21].

Highly complicated environmental and genetic factors are involved in the complex interplay controlling mandibular growth [22], such as heredity, rotation of the anterior cranial fossa, tooth eruption, pharyngeal growth, lip growth, cheeks and the tongue, changes in muscle behavior, nasal airway growth, changes in swallowing patterns, and bad oral habits. Karlsen believes that the shape of the mandible is related to growth type, the spatial direction of muscles, and the direction of the bite force [23]. The occlusal pressure produced by masticatory muscles affects the size and shape of the mandible, especially the length of the mandibular body and the height of the ramus [24]. Larger masseter and medial pterygoid muscles have a higher resting metabolic activity, keep bone under more tension, and grow in a more horizontal direction. As the mandibular angle increases, muscle metabolic activity decreases, and the decrease in bite force, muscle function, and biological efficiency might lead to decreased mandibular volume [25]. Furthermore, the mandibular growth direction is affected not only by the occlusal force but also by environmental factors such as orthodontic treatment, parafunctional habits, and functional malocclusion [26, 27].

This study showed that the mandibular ramus height, the mandibular body length, and the condylar width in skeletal class II patients were smaller than those in skeletal class I patients. The growth of the ramus height mainly depends on the new bone apposition in the mandibular condyle. The increase in mandibular body length is mainly due to the apposition of new bone on the lateral aspect of the mandible and the absorption of old bone on the medial aspect. The condyle undergoes cartilaginous growth. These findings suggest that mandibular condylar growth is insufficient, and mandibular body length is underdeveloped in skeletal class II patients.

In skeletal class II, GoR-Me-GoL and CoR-Me-CoL were larger than those in skeletal class I, indicating that the mandibular morphology was more stretched. According to the functional growth theory of the mandible, this change might be a kind of compensation. The growth of the mandible body length is restricted by the functional movements of the masseter, medial pterygoid, and temporalis muscles, relatively increasing bone deposition on the posterior part of the mandible, which decreases the curvature of the mandible.

Mandibular body height mainly depends on the growth of the alveolar process when the mandibular teeth erupt. The mandibular body height in skeletal class II patients is higher than that in skeletal class I patients, indicating that the mandibular anterior alveolar bone is overdeveloped, while the skeletal class II patients are prone to deep overbite. On the other hand, hypoplasia of mandibular body length leads to deep overjet. The anterior teeth do not contact, resulting in their supraspinatus and deep overbite.

Three-dimensional morphological differences of the mandible have their own special features in males and females. The mandibular body length and condyle width of skeletal class II male and female patients were smaller than those in skeletal class I male and female patients, respectively, consistent with the differences between the groups.

The mandibular ramus height and the condylar thickness in skeletal class II female patients were smaller than those in skeletal class I female patients, with no significant differences in these items in males. The growth of the mandibular ramus height ascending branch mainly depends on the apposition of the new bone in the mandibular condyle, which is the growth center of the mandible [28, 29]. The reason might be that the mandibular hypoplasia in females is more significantly affected by genetic factors, and condylar growth deficiency is responsible for this in males.

The opening of the mandibular body relative to Me and the mandibular body height in skeletal class II male patients were greater than those in skeletal class I male patients, with no significant difference in these items in females. The growth of the mandibular body height mainly depends on the increase in the alveolar process height during the eruption of the mandibular teeth [30]. The reason might be that mandibular hypoplasia in skeletal class II males is more significantly affected by environmental factors, and the compensation of muscles and the alveolar process is the main cause.

There was no significant difference in the distance between paired points, such as the width of the mandibular body and the distance between the sigmoid notch. There was no significant difference in the anteroposterior, mesial, and distal inclination of the ramus and condyle, indicating that the mandible in skeletal class II patients did not affect these items. Bayome et al. scanned the CBCT scans of 38 young adults with normal occlusion and found that the mandible in males was larger than that in females, but the mandibular angle in females was larger [20]. There is a moderate to strong correlation between several vertical and horizontal variables; for example, there is a negative correlation between condylar anteroposterior inclination and mandibular angle and a negative correlation between the ramus length and mandibular angle. It provides us with further research direction. The three-dimensional measurement of the mandibular shape and position should be further explored, and the pathogenic mechanism and morphological characteristics of skeletal class II patients should be studied to better guide the clinical diagnosis, scheme formulation, and prognosis evaluation in orthodontic treatment, orthognathic surgery, and other fields.

## 5. Conclusion

- There were statistically significant differences between skeletal class I and class II patients in the three-dimensional morphology of the mandible. The mandibular ramus height, mandibular body length, and condylar width in skeletal class II patients were smaller than those in skeletal class I patients. The mandibular body height in skeletal class II patients was higher than that in skeletal class I patients. The mandibular shape was more extended
- CBCT-assisted In Vivo Dental 5.1 software is a practical and effective method to study the three-dimensional morphology of the mandible. It can initially form a three-dimensional measurement method of the mandible, accurately describe the mandibular shape three-dimensionally, and guide clinical practice

## Figures and Tables

**Table 1:** The abbreviations and definitions of the landmarks.

Landmarks	Abbreviation	Definition
Coordinate system points		
Nasion	N	The most anterior point of the nasal frontal suture
Right porion	Po-R	The uppermost point of right external auditory canal
Right orbitale	Or-R	The lowest point of the right infraorbital margin
Anterior nasal spine	ANS	The apex of the anterior nasal ridge
Basion	Ba	The midpoint of the anterior edge of foramen magnum
Single points		
Sellar	S	The central point of the pituitary fossa
A	A	The most concave point of the bone between the point of anterior nasal ridge and the point of superior alveolar margin
B	B	The most concave point of the bone between the point of the inferior alveolar margin and the point of the anterior chin
Gnathion	Gn	The midpoint between the anterior point of chin and the submental point
Menton	Men	The lowest point of the chin
Ig	Ig	The apex of alveoli between lower central incisors
Paired points		
Left gonion	Go-L	Posterior inferior point of left mandibular angle
Right gonion	Go-R	Posterior inferior point of right mandibular angle
Internal point of left condyle	Co-L	The uppermost point of the left condyle
Internal point of right condyle	Co-R	The uppermost point of the right condyle
External point of left condyle	CdML	The innermost point of the left condyle
External point of right condyle	CdMR	The innermost point of the right condyle
Anterior point of left condyle	CdAL	The most lateral point of the left condyle
Anterior point of right condyle	CdAR	The most lateral point of the right condyle
Posterior point of left condyle	CdPL	The last point of the left condyle
Posterior point of right condyle	CdPR	The last point of the right condyle
Apex of left coracoid process	Cc-L	The uppermost point of the left coracoid process
Apex of right coracoid process	Cc-R	The uppermost point of the right coracoid process
Left sigmoid notch	Sg-L	The lowest point of the left sigmoid notch
Right sigmoid notch	Sg-R	The lowest point of the right sigmoid notch
Left mandibular inflection	Ma-L	The most concave and outermost point between left anterior edge of ramus and mandibular body turning point
Right mandibular inflection	Ma-R	The most concave and outermost point between right anterior edge of ramus and mandibular body turning point
Left mental foramen	Mf-L	The lateral superior point of the left mental foramen on the anterior surface of the mandible
Right mental foramen	Mf-R	The lateral superior point of the right mental foramen on the anterior surface of the mandible

**Table 2:** The line distance and angle measurements to describe the morphological characteristics of the mandible.

Mandibular body	

GoR-GoL	Mandibular body width
MfR-MfL	Width between mental foramen
Go-Ma (R/L)	Width between gonion
Go-Me (R/L)	Mandibular body length
Co-Gn (R/L)	Effective mandible length
Ma-Ig (R/L)	Alveolar process length
Ig-Men	Height of mandibular body
GoR-Me-GoL	The opening of mandibular body relative to Me
Co-Go-Me (R/L)	Mandibular angle
Cc-Ma-Ig (R/L)	Anterior angle of mandible

Ramus of mandible	

SgR-SgL	Width between sigmoid notch
MaR-MaL	Width between the mandibular inflection points
Co-Go (R/L)	Ramus height
CoR-Me-CoL	The opening of mandibular ramus relative to Me
Cc-Sg-Co (R/L)	Coracoid-sigmoid notch-condylar angle
CoGo-Sag (R/L)	Anteroposterior inclination of ramus
CoGo-Cor (R/L)	Mesial and distal inclination of ramus

Condyle	

CcR-CcL	Width between coracoid processes
CoR-CoL	Width between condylar
Co-Cc (R/L)	Distance between condyle and coracoid
CdM-CdD (R/L)	Condylar width
CdA-CdP (R/L)	Condylar thickness
Co-Sg (R/L)	Condylar height
CdMCdD-Sag (R/L)	Anteroposterior inclination of condyle
CdMCdD-Cor (R/L)	Mesial and distal inclination of condyle

**Table 3:** The difference between skeletal class I and skeletal class II ($\bar{x}\pm \text{s}$).

Measurement landmark	Skeletal class I	Skeletal class II	*P* value
Co-Go	61.080 ± 4.690	57.527 ± 5.857	0.012
Ig-Men	29.655 ± 2.073	31.049 ± 29.40	0.039
Go-Me	83.524 ± 4.242	80.312 ± 3.413	0.002
CdM-CdD	19.045 ± 2.051	17.135 ± 2.528	0.002
GoR-Me-GoL	67.779 ± 4.516	70.586 ± 4.368	0.017
CoR-Me-CoL	50.694 ± 2.951	52.439 ± 2.661	0.019

**Table 4:** The difference between skeletal class I and skeletal class II in male ($\bar{x}\pm \text{s}$).

Measurement landmark	Skeletal class I (male)	Skeletal class II (male)	*P* value
Go-Me	84.979 ± 4.339	81.526 ± 3.168	0.019
Ig-Men	30.599 ± 1.588	33.033 ± 2.654	0.005
CdM-CdD	20.086 ± 1.985	18.326 ± 2.346	0.035
GoR-Me-GoL	68.972 ± 4.252	73.081 ± 3.518	0.007

**Table 5:** The difference between skeletal class I and skeletal class II in female ($\bar{x}\pm \text{s}$).

Measurement landmark	Skeletal class I (female)	Skeletal class II (female)	*P* value
Go-Me	82.069 ± 3.729	79.099 ± 3.308	0.029
Co-Go	58.814 ± 2.599	53.324 ± 4.150	0.0001
CdM-CdD	18.005 ± 1.567	15.943 ± 2.165	0.006
CdA-CdP	10.567 ± 1.370	9.442 ± 1.401	0.034

## Data Availability

The data used to support the findings of this study are available from the corresponding author upon request.
